# Liposomal *N*-acylethanolamine-hydrolyzing acid amidase (NAAA) inhibitor F96 as a new therapy for colitis

**DOI:** 10.1039/d0ra05264g

**Published:** 2020-09-15

**Authors:** Yangyan Xiu, Kaizhen Wang, Jingfang Chen, Zhiqiang Zhuo, Yanghui Xiu

**Affiliations:** a Department of Gastroenterology and Respiration, Xiamen Branch of Children's Hospital of Fudan University (Xiamen Children's Hospital) 361006 China jfchen01@126.com; b Xiamen Huli District Maternity and Child Care Hospital 361006 China q661113@sina.cn; c Department of Infection, Xiamen Branch of Children's Hospital of Fudan University (Xiamen Children's Hospital) 361006 China; d Xiamen University Affiliated Xiamen Eye Center Xiamen Fujian 361006 China xiuyh1980@163.com; e Eye Institute of Xiamen University, School of Medicine, Xiamen University Xiamen Fujian 361006 China

## Abstract

Despite numerous advances in the pathological mechanism of inflammatory bowel disease (IBDs), the ideal therapy is still missing. *N*-Acylethanolamine-hydrolyzing acid amidase (NAAA), a cysteine hydrolase that deactivates fatty acid ethanolamides, has been recognized as a new therapeutic target for IBDs. Herein, we proposed liposomal F96, a selective and potent NAAA inhibitor, as a new therapy for IBDs. F96, with an IC_50_ of 270 nM for NAAA, was encapsulated into anionic liposome and the anti-inflammatory activity was evaluated in dextran sulfate sodium (DSS) induced colitis mice. The anionic liposomes showed significantly higher accumulation in the colon compared with the small intestine and cecum at 6 and 10 h after administration in DSS induced colitis mice. DSS induction significantly increased myeloperoxidase (MPO) activities and shortened the colon length, while free F96 significantly lowered tissue MPO activity and restored the colon length. Anionic liposome encapsulation significantly enhanced the therapeutic efficacy of F96, as liposomal F96 resulted in lower MPO activity and better colon length restoration effects compared with those treated with free F96. This study offers a new treatment option for colitis, which may pave the way for new therapies for other IBDs.

## Abstract 2

Liposomal NAAA inhibitor F96 exhibits potent therapeutic activities against colitis.

## Graphical abstract

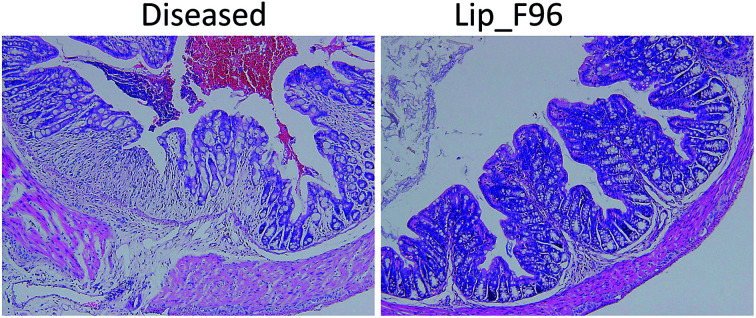

## Introduction

Inflammatory bowel diseases (IBDs), *e.g.*, colitis, are chronic inflammatory disorders of the gastrointestinal tract. Although IBDs are rarely lethal, these diseases can significantly impair patients' quality of life.^1^ The conventional therapeutic strategies for IBDs included classic anti-inflammatory compounds, such as 5-aminosalicylic acid derivatives, steroids, and immunoregulatory agents, such as azathioprine and methotrexate.^2,3^ Although those treatments alleviated inflammation in IBDs, these compounds either show serious side effects or induce dependence and tolerance. Biological therapeutic approaches, such as antitumor necrosis factor (TNF) antibody therapy, made remarkable progress in IBD therapy. Anti-TNF agents led to mucosal healing in a subgroup of IBD patients; however, many patients do not respond to anti-TNF treatment.^4^ New therapeutic targets and pharmacologic approaches are still highly desired for IBDs.

*N*-Acylethanolamine-hydrolyzing acid amidase (NAAA) is a lysosomal enzyme involved in degradation of endogenous fatty acid ethanolamides (FAEs), including palmitoylethanolamide (PEA). PEA activates the peroxisome proliferator-activated receptor (PPAR-a) and therefore reduces inflammation and pain.^5^ A recent study showed that pharmacological blockade of NAAA counteracts colitis by increasing colon PEA levels.^6^ F96, 3-(6-phenylhexanoyl)oxazolidin-2-one, is an oxazolidinone imide compound, which potently and selectively inhibited NAAA activity (IC_50_ = 270 nM).^7^ F96 exhibited potent analgesic activity in spared nerve injury mice model and alleviated lipopolysaccharide (LPS)-induced inflammation in mouse macrophage RAW264.7 cells.^8,9^ Those evidences suggest that F96 could also be a promising therapeutic agent for IBDs, *e.g.*, colitis, however, so far, no relevant studies have been reported.

As a lipophilic compound with a log *P* value of 3.08, F96 is expected to interact with glycoproteins and lipids in the intestinal mucus after oral administration, leading to limited drug amount in the epithelium layer to exhibit its pharmacological activity.^10^ A suitable delivery carrier is required to achieve potent *in vivo* anti-inflammatory effects after oral administration. Liposomal platforms have for many years been among the most promising nanocarriers for drug delivery and have been widely studied for the past few decades. Liposomes composed of one or more phospholipid bilayers are able to carry both hydrophilic and hydrophobic molecules due to their amphiphilic nature. They are biodegradable and biocompatible since they share similar compositions with biomembrane.^11^ The distribution of liposomes in gastrointestinal tract is largely dependent on the surface properties.^12,13^ It has been reported that cationic liposomes were preferably adhered to the healthy mucosa of the rat colon, while anionic liposomes largely accumulated in the inflamed mucosa.^14,15^ Those findings suggested that negatively charged liposomes could be an ideal drug carrier for the treatment of colitis, where inflammation is highly confined to the mucosal layer of the colon.^16^

**Scheme 1:**
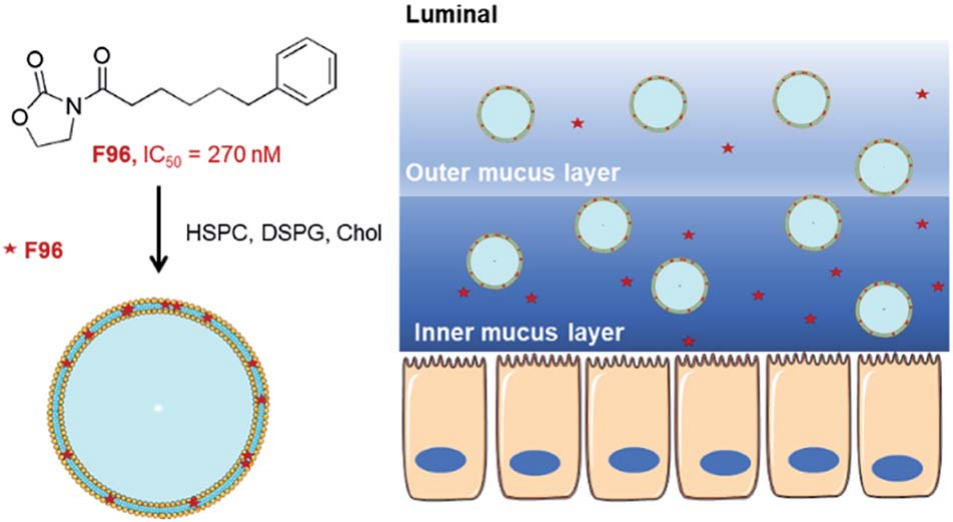
Delivery of liposomal *N*-acylethanolamine-hydrolyzing acid amidase (NAAA) inhibitor F96 for colitis treatment.

This study aimed to develop a highly efficient liposomal F96 formulation for the treatment of colitis. To the best of our knowledge, this is the first study using liposomal carrier to deliver NAAA inhibitor for the treatment of colitis. Our hypothesis is that the anionic liposomes would accumulate in the inflamed mucosa of the colon and slowly release the active compound F96 (Scheme 1). The released F96 would inhibit NAAA in the mucosa of the colon and increase local PEA levels, thus exhibited anti-inflammatory effects in colitis mice. As a proof of concept, the activity of anionic liposome encapsulated F96 against dextran sulfate sodium (DSS) induced colitis will be tested in the mice and compared with the anti-inflammatory activity of free F96. Inflammation severity will be assessed by monitoring tissue myeloperoxidase (MPO) activity, the weights of the dissected colons and hematoxylin and eosin (H&E) staining of colon tissues. The results suggest that anionic liposome significantly enhanced the therapeutic efficacy of F96, resulted in lower MPO activity and better colon length restoration effects. This study may offer a new safe and efficient therapeutic approach for inflammatory bowel diseases.

## Materials and methods

### Materials

F96 was synthesized following a previous reported method.^9^ Hydrogenated soybean phosphatidylcholine (HSPC) and 1,2-distearoyl-*sn-glycero*-3-phosphoglycerol, sodium salt (DSPG), and cholesterol were obtained from Lipoid (Ludwigshafen, Germany). Phosphate buffered saline tablets were from Sigma Chemical Co. (St Louis, MO, USA). 1,1′-Dioctadecyl-3,3,3′,3′-tetramethylindocarbocyanine perchlorate (Dil) was purchases from Thermo Fisher Scientific (Indianapolis, IN, USA). Dextran sulfate sodium (DSS) with an average molecular weight of 36 000–50 000 was purchased from MP Biomedicals, LLC (Solon, OH, USA). All other chemicals were of analytical grade unless otherwise stated in the text.

### Preparation and characterization of F96 encapsulated liposomes

Anionic liposome composed of HSPC : DSPG : cholesterol at a molar ratio of 5 : 1 : 4 were prepared by hydration of dry lipid film method. Briefly, all the three lipids were dissolved in chloroform and dried with N_2_ for several minutes, followed by overnight evaporation under vacuum. Dry lipid film was then dispersed in 10 mM phosphate buffered saline (PBS) at a concentration of 10 mg mL^−1^ and subsequently extruded through 200 and 100 nm polycarbonate membranes for 11 passages. F96 encapsulated liposomes were prepared by dissolved F96 in chloroform together with lipids and then following the same procedures as empty liposomes. F96 encapsulated liposomes samples was dialyzed against 10 mM PBS overnight to remove free F96.

The sizes of the liposomes, with and without encapsulated F96 were determined by dynamic light scattering measurement (Malvern ZetaSizer Nano ZS). The zeta-potential values were determined at pH 5.0, 6.5 and 7.5 (0.01 mol L^−1^ NaCl) at 25.0 °C by quasi-elastic light scattering analysis using the Malvern ZetaSizer Nano ZS. The morphology of F96 encapsulated liposomes was observed by cryo-transmission electron microscopy (cryo-TEM). A droplet of F96 liposomes at a lipid concentration of 100 μM was placed on a carbon support film, the excess liquid was blotted with a filter paper, and the grid was then plunged into a liquid ethane bath cooled with liquid nitrogen. Samples were maintained at a temperature of approximately −170 °C, using a cryo holder (Gatan), and were observed with a FEI Tecnai F20 electron microscope operating at 200 kV. The encapsulation efficiency and loading efficiency of F96 was determined by separation of free F96 *via* a using Amicon Ultra Centrifugal Filter Devices (Millipore, molecular weight cut-off, MWCO: 3k). F96 was quantified by high-performance liquid chromatography (HPLC) using a previously reported method.^9^

### *In vitro* F96 release study

F96 release from liposomes was investigated *in vitro* using dialysis membranes. Briefly, 4 mL of liposomes containing about 1 mg mL^−1^ F96 were placed in a dialysis bag (MWCO approximately 14 000, Sinopharm Chemical Reagent Co. Ltd, China). The dialysis bag was placed in a flask containing 100 mL release medium (PBS, pH 7.4, containing 0.2% Tween-80), and placed in a water bath shaking at 100 rpm at 37 °C. At predetermined time intervals, 1 mL of the release medium was withdrawn and replaced with an equal volume of fresh medium. The concentration of F96 in the medium was determined by high-performance liquid chromatographic (HPLC) and used to calculate the cumulative release percentage of F96.

### Cell toxicity of free F96 and liposomal F96 in Caco-2 cells

The cytotoxicity of free F96 and liposomal F96 on Caco-2 cells was evaluated using the 2,3-bis(2-methoxy-4-nitro-5-sulfophenyl)-5-[(phenylamino)carbonyl]-2*H*-tetrazolium hydroxide (XTT) method.^17^ Caco-2 cells were cultured in a 96-well plate and incubated for 24 h at 37 °C (1 × 10^4^ cells per well). The cells were treated with free F96 and liposomal F96 (0, 1, 2.5, 5, 10, 25, 50, 100, 200 μM) for 4 h and 12 h at 37 °C. The treatment media was then replaced by 100 μL of growth medium and cultured for 2 h and 10 h. XTT solution (25 μL) was then added to the cell medium and cells were cultured for another 2 h. The control cells were treated with medium alone without F96. The absorbance at 450 nm were measured using a microplate reader (Multiskan GO, China). Cell viability was calculated as a percentage to control cells.

### DSS-induced colitis mice model

All animal experiments were performed in accordance with Guide and Care and Use of Laboratory Animals from National Institutes of Health (NIH) and ARRIVE, and approved by the Animal Care and Use Committees of Xiamen University in China. To induce colitis, 5% DSS was added to the drinking water over 5 days, and this DSS containing solution was replaced with normal drinking water for an additional 2 days. Mice were sacrificed on day 7. According to previous report, colitis was established on day 5 based on weight loss and the presence of occult blood in the feces.^17^ Free F96 (10 mg kg^−1^), F96 encapsulated liposomes (10 mg kg^−1^) or empty liposomes was administered intragastrically once daily in the fed state, starting on day 5. Control mice received only normal drinking water. Body weight was monitored daily. Tissues were removed at the time of sacrifice and immediately frozen in liquid nitrogen. Before freezing, the length of colons was measured.

### Distribution of NPs in the gastrointestinal tract (GI) tract

To determine the distribution of anionic liposomes in the GI tract, anionic liposomes were labelled with a lipophilic carbocyanine dyes, 1,1′-dioctadecyl-3,3,3′,3′-tetramethylindocarbocyanine perchlorate (Dil). *In vivo* distribution of Dil labelled liposomes in the GI tract was evaluated using DSS-induced colitis mice. The experimental mice were fasted for 24 h prior to the administration of Dil liposomes. The Dil liposomes at a Dil dose of 1 mg kg^−1^ were administered by oral gavage under isoflurane aesthesia. After 2, 6 and 10 h, mice were sacrificed and all GI tract segments including luminal contents were collected and divided into 4 sections, including stomach, small intestine, cecum, and colon. The tissue samples were homogenized in PBS and then subsequently extracted with an ethanol/DMSO mixture (1 : 1 v/v). Specimens excised from the nontreated mice were also homogenized following the same protocol and used as blanks. The amount of Dil in the samples were then analysed using a fluorescence plate reader.

### Determination of myeloperoxidase (MPO) activity

The harvested tissue specimens were warmed to 37 °C, homogenized in 10 volumes of 0.02 M phosphate-buffer, pH 7.4, for the analysis of MPO activity. Briefly, 1 mL homogenized specimen was centrifuged at 20 000*g* for 10 min. The pellet was then resuspended in 0.5 mL ice cold 50 mM phosphate buffer (pH 6.0), containing 1 mL hexadecyltrimethyl ammonium bromide (HTAB). The suspension was freeze–thawed for two times, followed by sonication for 15 s and centrifugation at 25 000*g* for 5 min. The supernatant (0.1 mL) was then added to 2.9 mL of phosphate-buffer (pH 6), containing 0.167 mg mL^−1^ odianisidine hydrochloride and 5 × 10^−4^% v/v of hydrogen peroxide. The kinetics of absorbance change was determined at 460 nm over 30 s and slopes were calculated. The total protein levels of colon tissue were measured by the bicinchoninic acid assay (BCA assay), and the results of MPO activity were expressed per mg protein.

### Hematoxylin and eosin (H&E) staining of colon tissues

Distal parts of the colons from the healthy mice, and colitis mice with different treatments were first fixed by incubation with 4% (v/v) buffered formalin and 70% (v/v) alcohol, and then embedded in paraffin.^18^ Tissue sections of the distal colon were then prepared, stained with H&E, followed by imaged using a light microscope (Zeiss, Axioskop, Germany).

### Statistical analysis

All data were presented as the mean ± standard deviation of three or four independent experiments. All statistical analyses were completed using the Students' *t*-test in GraphPad Prism (version 5.01), and *p* < 0.05 was considered statistically significant.

## Results

### Preparation and characterization of liposomal F96

**Fig. 1:**
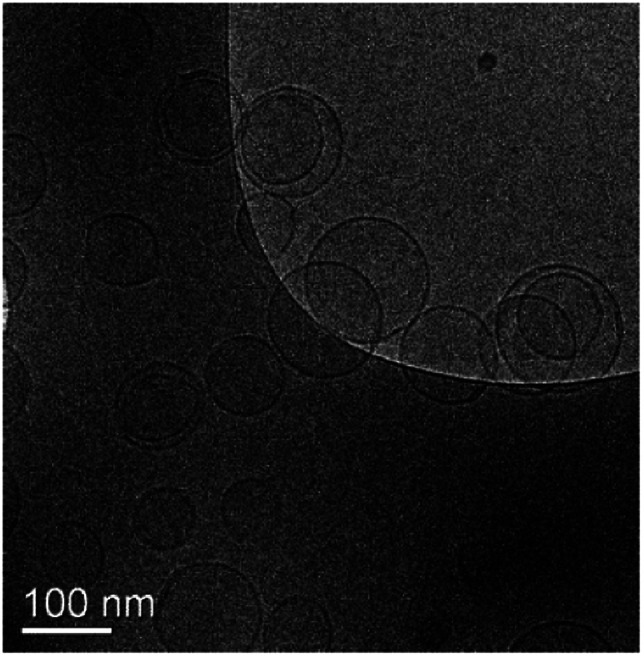
A representative cryo-TEM image F96 encapsulated liposomes.

F96 encapsulated anionic liposomes were obtained by the thin film hydration method. As shown in Table 1 empty liposomes had an average hydrodynamic diameter of 115 nm with a polydispersity of 0.115. F96 encapsulated liposomes showed negative zeta potential of approximately −32, −40 and −48 mV at pH 5.0, 6.5 and 8.0, respectively. The incorporation of F96 into the liposomes did not have a distinct effect on particle size and zeta potential. The encapsulation efficiency of F96 in anionic liposomes is over 88% and the loading efficiency is around 5.6%. Most of F96 liposomes show unilamellar and circular structure, while few of them exhibited multilamellar structure as shown in the cryo-TEM image (Fig. 1).

**Table 1:** Physicochemical characteristics of liposomes^a^

	Size (nm)	PDI	EE (%)	LD (%)
Blank Lip	115 ± 20	0.115 ± 0.043	—	—
F96 Lip	118 ± 12	0.085 ± 0.020	88.6 ± 8.2	5.6 ± 0.4

a Lip, liposomes; PDI, polydispersity index; EE, encapsulation efficiency; LE, loading efficiency.

### *In vitro* liposome stability and F96 release profile

**Fig. 2:**
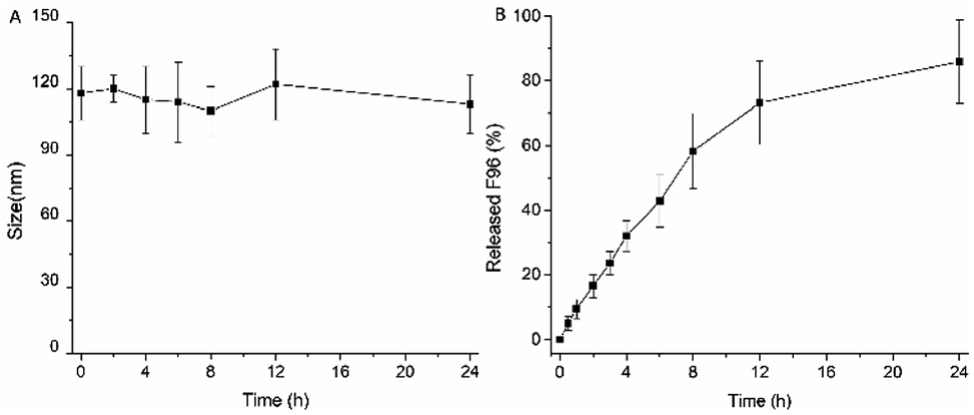
Liposome size stability (A) and *in vitro* release profiles (B) of F96 from anionic liposomes.

The particle size of F96 encapsulated liposomes remained unchanged upon incubation with PBS (pH 7.4) under 37 °C in 24 h (Fig. 2A). *In vitro* F96 release from liposomes was evaluated at a pH value of 7.4, which resemble the colon pH value. Tween-80 (0.2% w/v) was added to the release medium to maintain a sink condition during the release study. As shown in Fig. 2B, no burst release was observed for the F96 encapsulated liposomes, suggesting the effective incorporation of F96 within liposomes. The cumulative release percentage of F96 from liposomes was 85.9% over 24 h.

### Cell toxicity of free F96 and liposomal F96 in Caco-2 cells

**Fig. 3:**
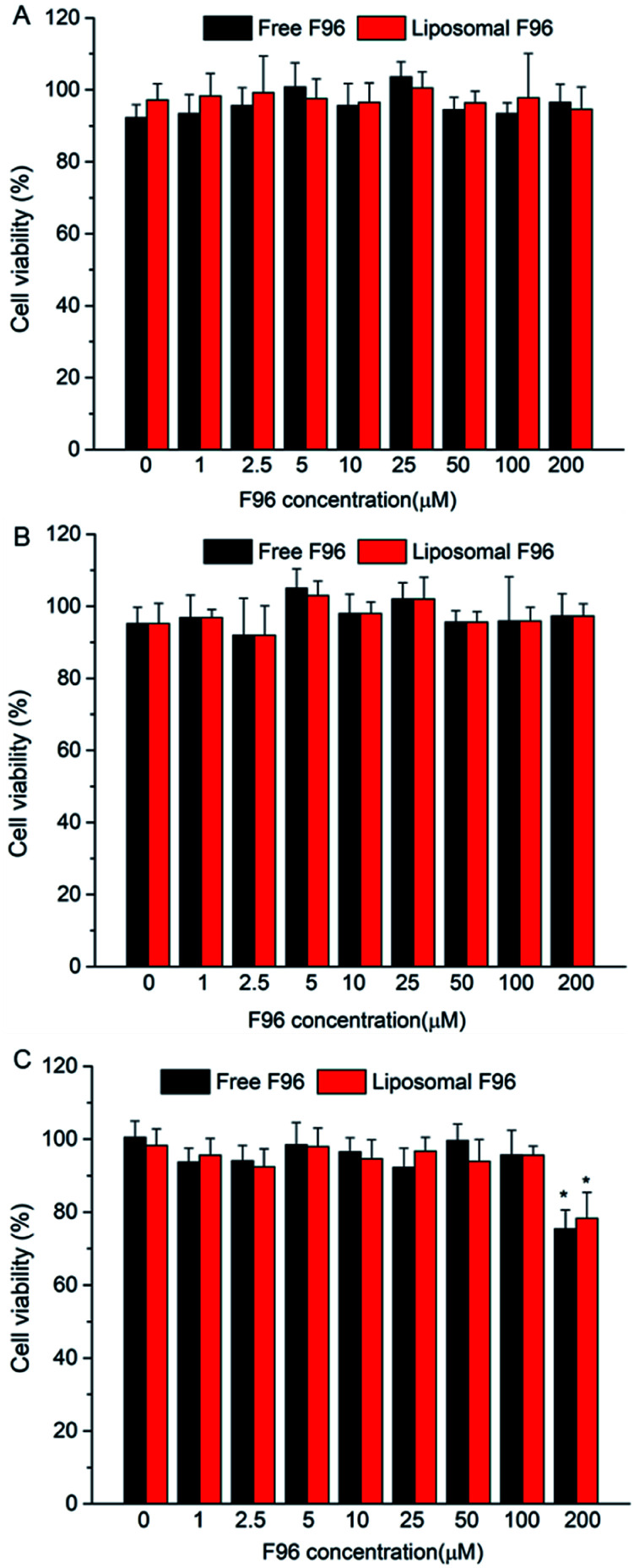
Cell toxicity of free F96 and liposomal F96 tested under different conditions. (A) Tested 4 h after 4 h incubation; (B) tested 12 h after 4 h incubation; (C) tested 12 h after 12 h incubation. *, *P* < 0.05, compared with Caco-2 cells treated with blank DMEM medium.

The cytotoxicity of free F96 and liposomal F96 are showed in Fig. 3. No significant cytotoxicity towards Caco-2 cells was observed for free F96 and liposomal F96 at the tested concentrations (0, 1, 2.5, 5, 10, 25, 50, 100, 200 μM) after 4 incubation tested 4 h and 12 h after treatment. However, both free F96 and liposomal F96 showed some toxicity at 200 μM upon 12 h incubation.

### *In vivo* distribution of F96 in the mice GI tract

**Fig. 4:**
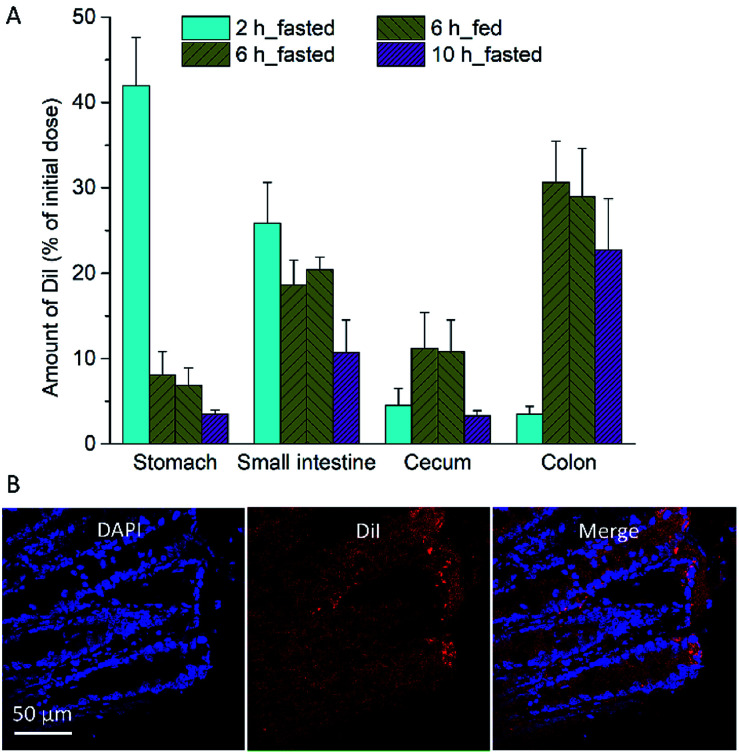
*In vivo* distribution of anionic liposomes in the different GI segments. (A) Quantification of Dil liposome in stomach, small intestine, cecum and colon at 2, 6, 10 h after administration in the fasted state. (B) Representative CLSM image of Dil liposome distribution in colon mucosa. Results are shown as three experiments ± SEM.

We then determined liposome distribution in the GI tract of DSS-induced colitis mice (Fig. 4). The percentage dose of Dil in each GI tract segment (stomach, small intestine, cecum, and colon) was measured at 2, 6, and 10 h after oral administration of Dil liposomes. As shown in Fig. 4A. Dil levels in the colon were significantly higher than that in small intestines and cecum at 6 and 10 h after administration. Confocal imaging of sections from colon tissue showed distribution of Dil liposomes in the crypt (Fig. 4B). When used as a delivery carrier, this anionic liposome is promising to enhance the therapeutic efficacy of F96.

### *In vivo* anti-inflammatory studies

**Fig. 5:**
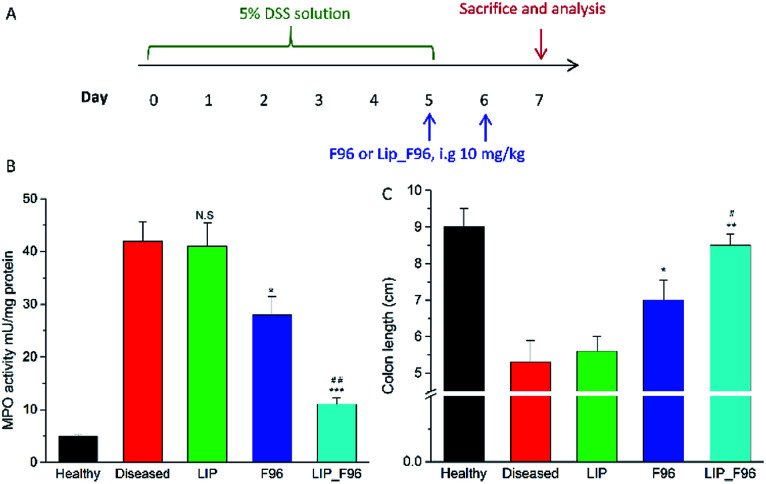
MPO activity and length of colons. (A) Experimental timeline. (B) MPO activity in the colons of DSS induced colitic mice treated with saline (“Diseased”), blank liposome (LIP), free F96, and F96 encapsulated anionic liposomes (“LIP_F96”). (C) Length of colons mice with different treatment. Results are shown as four experiments ± SEM.*, *P* < 0.05; **, *P* < 0.01; ***, *P* < 0.001 compared with diseased control. ^#^, *P* < 0.05; ^##^, *P* < 0.01 compared with free F96 treated group.

We measured the activity of colonic MPO, the most abundant protein in neutrophils and widely used in IBD animal models and in feces of IBD patients as a standard indicator of colitis.^19^ The experimental timeline was showed in Fig. 5A. One day 5 after DSS induction, treated mice exhibited increased MPO activity compared with healthy controls (Fig. 5B). Free F96 treatment significantly lower the MPO activity in the colitis mice as compared with those in the untreated colitis mice. Liposomal F96 further lower the MPO activity compared with free F96 (*P* < 0.001), while blank liposomes didn't show any effects on the MPO activity, suggesting that the decreased MPO activity was caused by F96 released from liposomes. DSS exposure for 5 days also shortened the colon length of mice, while treatment with free F96 partially restored the colon length and liposomal F96 showed a stronger effect on the colon length (Fig. 5C).

**Fig. 6:**
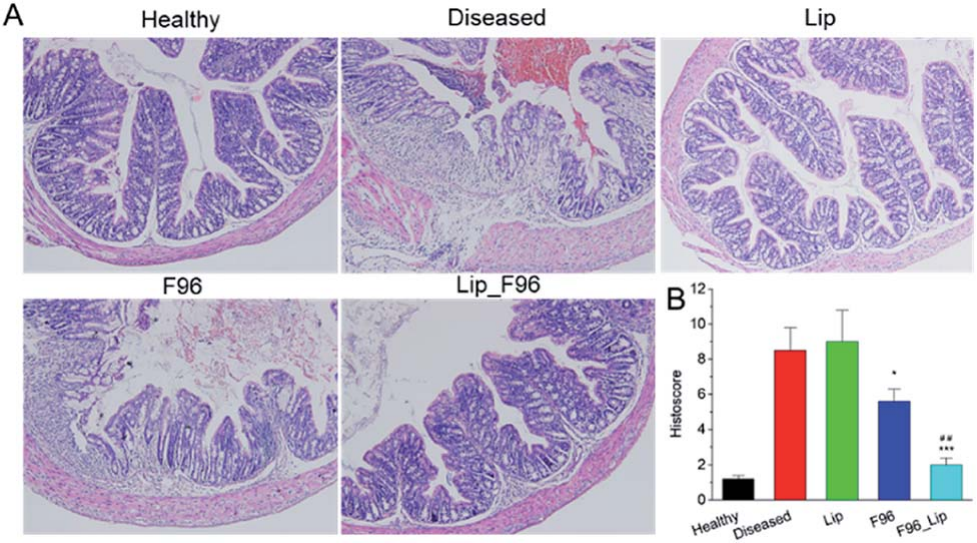
Representative histology staining of colon (A) and histological score (B).

H&E staining of distal colon showed that DSS induction resulted in colitis exhibiting epithelial degeneration, crypt loss and inflammatory cell infiltration (Fig. 6). Treatment with free F96 alleviated DSS-induced mucosal damage of colon. Liposomal F96 showed stronger effects towards restoring mucosal damage compared with free F96. Only minor mucosal damage was observed after treating colitis mice with liposomal F96. The results suggested that liposome encapsulation of F96 enhanced therapeutic potency of F96 towards colitis.

## Discussion

Despite significant advances in treatment of IBDs, these diseases remain a clinical problem, since there is no drug that works for most of patients. New therapeutic targets and agents for IBDs are still highly desired. NAAA, a lysosomal enzyme that involves in degradation of endogenous lipids, including PEA, has recently been identified as a new therapeutic target for colitis.^6^ PEA is an anti-inflammatory compound that activates peroxisome proliferator-activated receptor-α-dependent (PPAR-α) and exhibit beneficial effects on inflammations.^1,20^ F96 is a NAAA inhibitor that selectively and potently inhibit NAAA, lead to anti-inflammatory effects in many different inflammation experimental models.^7^ We used undifferentiated Caco-2 cells to examine the toxicity where F96 did not show significant toxicity. However, the result might not reflect what would happen in fully differentiated intestinal cells, since differentiation process strongly alters gene expression. As a lipophilic drug molecule, F96 is expected to be trapped in the intestinal mucus after oral administration, leading to limited drug amount in the epithelium layer to exert its pharmacological activity. Therefore, suitable drug carriers are required to achieve high potent anti-inflammatory effects towards IBDs.

In this study, we first demonstrate the anti-inflammatory activities of F96 DSS induced colitis mice. DSS induction significantly increased myeloperoxidase (MPO) activities and shorten the colon length. Free F96 significantly lowered tissue MPO activity, restored the colon length, and alleviated DSS-induced mucosal damage of colon tissue. These results suggested that F96 is a promising therapeutic agent for colitis. Encapsulation of F96 into anionic liposome significantly enhanced the therapeutic efficacy of F96, as lower MPO activity, better colon length restoration effects and less mucosal damage were observed. It has been previously reported that anionic liposomes tended to accumulated in the inflamed colon,^14^ which may enhance the anti-inflammatory activities of F96 towards colitis. This is a strong evidence showed that a combination of new therapeutic agents and drug carrier may lead to better therapeutic effects. Currently there are few researches have been reported in this field. Close collaborations between scientists from pharmaceutical chemistry and pharmaceutical science may pave the way towards new treatments for IBDs.

## Conclusion

To improve the therapeutic potency of NAAA inhibitor F96 for colitis, liposomal F96 was developed and evaluated in DSS-induced colitis mice. Free F96 ameliorated DSS-induced colitis, lowered the MPO activity and restored mucosal damage. Encapsulation of F96 by anionic liposome significantly enhanced its beneficial effects towards colitis. The results suggested that this liposomal F96 is a potential therapy for colitis and it may also work for other IBDs. This study suggested customizing drug carriers for highly potent drug candidates may pave the way towards new potent therapeutic approaches.

## Conflicts of interest

The authors claim no conflicts of interest.

## Supplementary Material
